# Patterns and correlates of sedentary behaviour among people with multiple sclerosis: a cross-sectional study

**DOI:** 10.1038/s41598-021-99631-z

**Published:** 2021-10-13

**Authors:** Jennifer Fortune, Meriel Norris, Andrea Stennett, Cherry Kilbride, Grace Lavelle, Wendy Hendrie, Christina Victor, Jennifer Mary Ryan

**Affiliations:** 1 Department of Public Health and Epidemiology, RCSI University of Medicine and Health Sciences, Dublin, Ireland; 2 Ageing Studies Theme, Institute of Environment, Health and Societies, Brunel University London, London, UK; 3 Wolfson Institute of Preventive Medicine, Queen Mary University of London, London, UK; 4 Institute of Psychiatry, Psychology and Neuroscience, King’s College London, London, UK; 5 MS Therapy Centre, Norwich, UK

**Keywords:** Multiple sclerosis, Lifestyle modification, Rehabilitation

## Abstract

High levels of sedentary behaviour are associated with poor health outcomes in people with multiple sclerosis (MS). Identifying modifiable correlates of sedentary behaviour for people with MS is essential to design effective intervention strategies to minimise sedentary time. This study aimed to quantify patterns and identify correlates of sedentary behaviour among adults with MS. Fatigue, self-efficacy, walking capability, the physical and psychological impact of MS, health-related quality of life, and participation and autonomy were assessed by questionnaire. Participants wore an activPAL monitor. Total (min/day), prolonged bouts (≥ 30 min) and breaks in sedentary time were calculated. Associations were examined using regression analysis adjusted for demographic and clinical confounders. Fifty-six adults with MS participated (mean ± SD age: 57.0 ± 9.25 years; 66% female). Self-efficacy for control over MS was associated with sedentary time (β = 0.16, 95% CI 0.01, 0.30). Self-efficacy in function maintenance (β = 0.02, 95% CI 0.00, 0.04), health-related quality of life (EuroQol-5D) (β = 31.60, 95% CI 7.25, 55.96), and the autonomy indoors subscale of the Impact on Participation and Autonomy Questionnaire (β = − 5.11, 95% CI − 9.74, − 0.485) were associated with breaks in sedentary time. Future studies should consider self-efficacy, health-related quality of life and participation and autonomy as potential components of interventions to reduce sedentary behaviour.

## Introduction

Sedentary behaviour (SB) is defined as any waking behaviour undertaken in a sitting, lying, or reclining posture that requires no more than 1.5 metabolic equivalents of energy expenditure^1^. SB is linked to negative health outcomes including premature mortality, cardiovascular disease, type 2 diabetes, cancer and obesity^2^. Crucially, the hazards of SB appear most pronounced in physically inactive populations^2^.

People with multiple sclerosis (MS) are less physically active and demonstrate higher levels of SB than the general population^3^. In people with MS, SB is associated with higher levels of disability, slower walking speed and lower endurance^4^, comorbid conditions such as hypertension ^5^, and secondary complications including spasms, pain and reduced skin integrity that can compound primary MS symptoms^6^. Additional to total time in SB, the pattern of accumulation may influence health outcomes^7^. Prolonged bouts of sedentary time are associated with higher mortality ^8^ and deleterious effects on cardiometabolic health in the general population^9,10^. Furthermore, frequent interruptions to sedentary time demonstrate beneficial effects on cardiometabolic risk^11,12^. Accordingly, there has been increasing interest in reducing SB and modifying accumulation patterns as a preventative approach to improve health and manage MS-related symptoms. Understanding the association between specific determinants and sedentary outcomes in people with MS may provide a theoretical underpinning to guide and inform intervention approaches to reduce sedentary behaviour.

Previous studies have found that demographic and clinical characteristics such as MS type, duration, and disability status are related to self-reported sedentary time^13,14^. However, self-reported measures significantly underestimate sedentary time compared to device measures ^15^ and typically provide estimates of volume, but not patterns of SB. Similarly, studies examining objective SB have reported age, MS type, disease duration, disability status via the Patient Determined Disease Steps (PDDS) scale ^16^ and fatigue ^17^ as correlates, with more recent research showing associations with Social Cognitive Theory constructs^18^. However, these studies used hip-mounted accelerometers, which characterise sedentary behaviour through periods of inactivity measured by count-based movement thresholds (e.g. < 100 counts per min)^16,18^. Since movement is determined by acceleration rather than body posture they cannot robustly differentiate between sitting and upright positions and may misclassify static postures such as standing as sedentary behaviour^19^. Differentiating between standing, sitting and lying may be particularly important for people with mobility impairment as activities in standing may require significant energy expenditure. Moreover, hip-based accelerometers typically rely on waking hour rather than 24-h measurement protocols and require removal and reattachment for sleeping, showering and aquatic activities. Premature removal or failure to reattach accelerometers may lead to an underestimation of SB. Indeed, SB estimates are more affected by non-wear time compared to physical activity^20^.

Thigh worn inclinometers overcome the limitations of hip-mounted accelerometers by directly quantifying postures ^21^ and are often considered the gold standard for the objective measurement of volume and patterns of SB^22^. One study has explored sedentary behaviour outcomes in people with MS using this recommended measurement tool^23^. To our knowledge, correlates of inclinometer-measured sedentary time and patterns of sedentary time have not been explored in people with MS. This study aimed to quantify patterns of SB among community-dwelling people with MS using a thigh worn inclinometer and identify correlates of SB.

## Methods

This study was a cross-sectional analysis of baseline data from the iStep-MS trial, a feasibility randomised controlled trial of a behaviour change intervention, which aimed to increase physical activity and reduce SB in people with MS^24^.

### Participants

People with MS were recruited from an MS Therapy Centre in England and the MS Society UK website. Inclusion criteria were a self-reported diagnosis of MS, ability to independently walk within the home with or without a walking aid, relapse-free for three months, and free of unstable medical conditions that would make it unsafe to participate in physical activity. Exclusion criteria were pregnancy and ongoing participation in other trials. The College of Health and Life Sciences Research Ethics Committee in Brunel University London (6181-NHS-Apr/2017-7016-2) approved this study. All research was performed in accordance with the Declaration of Helsinki. Informed consent was obtained from all participants.

### Sedentary behaviour

Sedentary behaviour was assessed using the activPAL activity monitor (PAL Technologies, Glasgow, UK). The activPAL was waterproofed, attached on the midline, anterior aspect of the upper thigh using a Hypafix dressing and worn 24 h day^−1^. Data were processed in Stata (StataCorp LP, College Station, Texas) using a validated automated algorithm^25^ to separate valid waking wear data from time in bed, non-wear data and invalid data. Heatmaps were created to visually inspect the processed valid and invalid data. Where the algorithm appeared to incorrectly code data as valid/invalid, activity diaries were checked against the heat maps and data were corrected if necessary. Data were considered valid if a day consisted of ≥ 10 h of waking wear data^25^. Participants were required to have at least 2 valid days to be included in the analysis^26^. After identification of valid waking wear data, the following outcomes were calculated: (1) total sedentary time (sitting/lying time in minutes); (2) number of prolonged bouts of sedentary time (sitting/lying bouts lasting ≥ 30 min); (3) number of breaks in sedentary time (defined as a transition from sitting or lying to an upright posture); (4) time in moderate to vigorous physical activity (MVPA) (in min). Sedentary behaviour outcomes and MVPA were averaged over the number of valid wear days.

### Independent variables

Fatigue was assessed using the Modified Fatigue Impact Scale (MFIS); higher scores indicated greater impact of fatigue on activities. Self-efficacy was assessed using the Multiple Sclerosis Self-Efficacy Scale (MSSE); higher scores indicated greater self-efficacy. Walking capability was assessed using the 12-item MS Walking Scale (MSWS-12); higher scores indicated poorer walking capability. The physical and psychological impact of MS was assessed using the Multiple Sclerosis Impact Scale (MSIS-29); higher scores indicated greater disease impact on daily function. Health-related Quality of life (HRQOL) was assessed using EuroQol-5D-5L (EQ-5D-5L). The United Kingdom value set was used to calculate a utility score ^27^. Participation and autonomy over four domains (autonomy indoors, family role, autonomy outdoors, social life and relationships) was assessed using the Impact on Participation and Autonomy Questionnaire (IPA). The median score was obtained for each participant for each subscale. A detailed description of the measurement of these variables is provided elsewhere^28^. Variable scoring is outlined in Supplementary Table 1.

### Demographic and clinical confounders

The following variables were considered as potential confounders: age (years); body mass index (BMI: kg/m^2^), sex (male, female), ethnicity (White, Black, Asian), living arrangement (living alone, living with family/partner), employment (employed, not employed), marital status (married/partnered, not married/partnered), MS type (relapsing–remitting, secondary progressive, primary progressive or unknown), disease duration (years), disability status (Expanded Disability Status Scale (EDSS) 1.0–4.0 or 4.5–6.5) and falls history (non-fallers i.e. no self-reported falls in preceding 12 months, or fallers i.e. ≥ 1 falls in previous 12 months).

### Data analysis

Statistical analyses were performed using Stata, version 16.0. Data distribution was examined using histograms, Q–Q plots and cross-tabulations. Data are summarized as mean, standard deviation, median, minimum, maximum, frequencies and proportions as appropriate. Regression analysis was used to examine (1) the associations between demographic and clinical characteristics and SB outcomes (2) the associations between each SB outcome (as the dependent variable) and each independent variable. Potential confounding variables were added to each regression model one at a time and included in the final adjusted model if they modified the regression coefficient for the independent variable by > 10%. Interaction terms between the independent variable and EDSS category were separately added to the final models to examine whether the associations between the independent variable and SB outcome were modified by disability status. Finally, as there is mixed evidence that correlates of sedentary behaviour are independent of MVPA in the general population ^29^, MVPA was added to the final model to assess if correlates of sedentary behaviour in people with MS were independent of MVPA.

## Results

Sixty people with MS were recruited. Fifty-six participants were included in the analysis. Three participants did not return their monitor and data from another did not meet the analysis validity criteria. Table 1 displays participant characteristics. Participants had a mixed presentation of MS type and were predominantly female, white and classified in EDSS score subgroup 4.5–6.5.

**Table 1:** Participant characteristics.

	n (%)	Mean (SD)	Range
Age (years)	56	57.0 (9.25)	37–74
Women	37 (66)		
**Ethnicity**			
White	50 (89.3)		
Black	3 (5.36)		
Asian	3 (5.36)		
**Living arrangement**			
Lives alone	6 (10.71)		
Lives with partner/spouse/family member	50 (89.29)		
**Employment status**			
Employed	19 (33.93)		
Not employed	37 (66.07)		
**Marital status**			
Married/partnered	45 (80.36)		
Not married/partnered	11 (19.64)		
BMI (kg m^2^)	56	25.9 (4.72)	16.7–39.5
MS duration (years)	55	15.54 (9.95)	1–42
**Falls history**			
0 falls in previous 12 m	16 (28.57)		
> 0 falls in previous 12 m	40 (71.43)		
**Type of MS**			
Relapsing–remitting	19 (34)		
Secondary progressive	20 (36)		
Primary progressive	13 (23)		
Unknown	4 (7)		
**EDSS**			
1.0–4.0	15 (27)		
4.5–6.5	41 (73)		

*SD* standard deviation, *BMI* body mass index, *MS* multiple sclerosis, *EDSS* Expanded Disability Status Scale.

Participants wore the activPAL for a mean ± SD 905.4 ± 71.4 min/day (range 713.3–1040.7 min/day). Sedentary time, bouts and breaks in sedentary time, and MFIS, MMSE, MSIS, EQ-5D-5L and IPA scores are described in Table 2.

**Table 2:** Descriptive statistics of all variables.

	n = 56 Mean (SD)
Average sedentary time (min/day)	604.47 (107.55)
Average sedentary time as % of waking wear time (%)	67.07 (12.94)
Average prolonged bouts (≥ 30 min) of sitting/lying (n/day)	5.90 (1.67)
Average number of breaks in sitting per day (n/day)	49.58 (17.72)
Average MVPA time (min/day)	32.46 (28.03)
MFIS cognitive subscale (score 0–40)	16.88 (9.80)
MFIS physical subscale (score 0–36)	22.01 (8.12)
MFIS psychosocial subscale (score 0–8)	3.83 (2.10)
MFIS total score (score 0–84)	42.73 (18.26)
MMSE control subscale (score 90–900)	579.64 (200.19)
MMSE function subscale (score 90–900)	661.96 (201.08)
MSWS-12 total score (%)	74.37 (20.20)
MSIS-29 physical (0–100)	42.63 (21.55)
MSIS-29 psychological (0–100)	30.55 (19.12)
EQ-5D-5L utility	0.63 (0.19)
IPA: autonomy indoors (score 0–4)	0.64 (0.86)
IPA: family role (score 0–4)	1.32 (0.95
IPA: autonomy outdoors (score 0–4)	1.51 (1.07)
IPA: social life and relationships (score 0–4)	0.46 (0.60)

*MVPA* moderate to vigorous physical activity, *MFIS* Modified Fatigue Impact Scale, *MSSE* Multiple Sclerosis Self-Efficacy Scale, *MSWS-12* Twelve Item MS Walking Scale, *MSIS* Multiple Sclerosis Impact Scale, *EQ-5D-5L* EuroQol-5D-5L, *IPA* Impact on Participation and Autonomy Questionnaire, *SD* standard deviation.

Table 3 presents the associations between demographic and clinical characteristics and SB outcomes. People with secondary progressive and primary progressive MS spent more time in sedentary behavior than those with relapsing remitting MS. People with secondary progressive MS also had more prolonged bouts and fewer breaks in sedentary time than those with relapsing–remitting MS (p = 0.007 and p = 0.039). Participants of Asian ethnicity had fewer breaks in sedentary time than White participants (p = 0.039). No other associations were demonstrated. Sedentary outcomes based on demographic and clinical characteristics are described in Supplementary Table 2.

**Table 3:** Associations between demographic and clinical characteristics and sedentary behaviour outcomes.

	Sedentary time, (min/day)		Average prolonged bouts (≥ 30 min) of sitting/lying (n/day)		Average number of breaks in sitting per day (n/day)	
	Unadjusted β (95% CI)	p value	Unadjusted β (95% CI)	p value	Unadjusted β (95% CI)	p value
Age (years)	0.16 (− 3.01 to 3.33)	0.922	0.00 (− 0.05 to 0.05)	0.989	− 0.04 (− 0.56 to 0.48)	0.882
Sex	− 6.30 (− 67.69 to 55.09)	0.838	− 0.34 (− 1.29 to 0.61)	0.478	8.82 (− 1.01 to 18.65)	0.078
**Ethnicity**^**a**^						
Black	41.64 (− 85.59 to 168.86)	0.514	0.22 (− 1.78 to 2.22)	0.826	− 5.79 (− 26.43 to 14.84)	0.576
Asian	101.23 (− 25.99 to 228.45)	0.116	1.22 (− 0.78 to 3.22)	0.227	− 21.74 (− 42.38 to − 1.10)	0.039
Living arrangement	45.45 (− 47.75 to 138.64)	0.333	0.57 (− 0.88 to 2.02)	0.436	− 0.61 (− 16.10 to 14.88)	0.938
Employment status	− 8.71 (− 70.08 to 52.66)	0.777	− 0.28 (− 1.23 to 0.68)	0.562	5.64 (− 4.36 to 15.64)	0.263
Marital status	22.20 (− 50.74 to 95.14)	0.544	0.42 (− 0.71 to 1.55)	0.462	− 1.34 (− 13.39 to 10.72)	0.825
BMI (kg m^2^)	1.36 (− 4.84 to 7.55)	0.663	0.01 (− 0.09 to 0.11)	0.844	− 0.42 (− 1.44 to 0.59)	0.407
MS duration (years)	1.57 (− 1.41 to 4.54)	0.295	0.04 (− 0.01 to 0.08)	0.112	− 0.39 (− 0.88 to 0.09)	0.107
Falls history	9.49 (− 54.83 to 73.80)	0.769	0.13 (− 0.87 to 1.13)	0.799	− 6.11 (− 16.59 to 4.36)	0.247
**Type of MS**^**b**^						
SPMS	92.54 (26.97 to 158.10)	0.007	1.14 (0.08 to 2.20)	0.036	− 11.36 (− 22.60 to − 0.12)	0.048
PPMS	77.78 (4.12 to 151.45)	0.039	0.64 (− 0.55 to 1.83)	0.287	− 2.72 (− 15.35 to 9.91)	0.667
Unknown	27.33 (− 85.25 to 139.92)	0.628	0.84 (− 0.98 to 2.66)	0.357	− 2.09 (− 21.39 to 17.21)	0.829
EDSS	0.36 (− 0.66 to 1.38)	0.480	0.36 (− 0.66 to 1.38)	0.480	− 5.51 (− 16.22 to 5.21)	0.308

^a^Reference group = white.

^b^Reference group = relapsing remitting MS.

### Sedentary time

No associations with sedentary time (min/day) were demonstrated (Table 4). After adjustment for confounders, the MMSE control subscale was associated with sedentary time (β = 0.16, 95% CI 0.01, 0.30, p = 0.042). A one-unit increase in MMSE control score, indicating greater confidence to manage disease symptoms, reactions and impact on daily life, was associated with an additional 1.6 min/day of sedentary time. This association remained after controlling for MVPA (β = 0.16, 95% CI 0.02, 0.30, p = 0.023). After adjusting for confounders, there was also weak evidence of an association between sedentary time and MFIS total score (β = − 1.53, 95% CI − 3.08 to 0.02, p = 0.053).

**Table 4:** Associations between independent variables and sedentary time.

Dependent variable: sedentary time (min/day)	Unadjusted β (95% CI)	p value	Adjusted β (95% CI)	p value
**MFIS cognitive**				
Adjusted for EDSS, ethnicity, marital status, MS duration	− 1.31 (− 4.29 to 1.66)	0.380	− 2.66 (− 5.89 to 0.57)	0.104
**MFIS physical**				
Adjusted for ethnicity, living arrangement, MS type	− 0.17 (− 3.78 to 3.44)	0.380	− 3.30 (− 6.91 to 0.30)	0.072
**MFIS psychological**				
Adjusted for EDSS, employment, ethnicity living arrangement, marital status, MS duration	2.68 (− 11.30 to 16.66)	0.925	− 1.80 (− 18.80 to 15.21)	0.833
**MFIS total**				
Adjusted for ethnicity, living arrangement, MS type	− 0.38 (− 1.98 to 1.23)	0.702	− 1.53 (− 3.08 to 0.02)	0.053
**MSSE function**				
Adjusted for EDSS, ethnicity, MS type	− 0.11 (− 0.26 to 0.03)	0.640	0.01 (− 0.17 to 0.19)	0.913
**MSSE control**				
Adjusted for ethnicity, living arrangement, MS type	0.02 (− 0.13 to 0.16)	0.112	0.16 (0.01 to 0.30)	0.042
**MSWS-12**				
Adjusted for employment, ethnicity, falls history, MS type	1.11 (− 0.31 to 2.53)	0.822	0.10 (− 2.00 to 2.20)	0.923
**MSIS-29 psychological**				
Adjusted for ethnicity, marital status, MS type, sex	0.15 (− 1.38 to 1.69)	0.124	− 0.95 (− 2.56 to 0.66)	0.242
**MSIS-29 physical**				
Adjusted for EDSS, ethnicity, living arrangement, MS type	0.79 (− 0.56 to 2.13)	0.842	− 0.42 (− 2.06 to 1.23)	0.614
**EQ-5D-5L utility**				
Adjusted for EDSS, ethnicity MS type	− 116.55 (− 265.67 to 32.57)	0.245	40.60 (− 201.50 to 120.30)	0.614
**IPA: autonomy indoors**				
Adjusted for EDSS, ethnicity, MS type	26.60 (− 6.67 to 59.86)	0.115	3.39 (− 34.33 to 41.11)	0.857
**IPA: family role**				
Adjusted for ethnicity, marital status, MS duration, MS type	11.03 (− 19.53 to 41.59)	0.472	− 12.58 (− 45.12 to 19.96)	0.441
**IPA: autonomy outdoors**				
Adjusted for EDSS, ethnicity, living arrangement, marital status, MS duration, MS type	8.99 (− 18.10 to 36.09)	0.509	− 11.75 (− 45.21 to 21.71)	0.483
**IPA: social life and relationships**				
Adjusted for ethnicity, MS type, MS duration	35.41 (− 12.37 to 83.18)	0.143	14.85 (− 35.35 to 65.04)	0.555

There was evidence, as indicated by the p-value for the interaction term, that EDSS score modified the association between the MFIS physical subscale and sedentary time (p = 0.018) and the association between the MFIS total score and sedentary time (p = 0.030). The MFIS physical subscale and MFIS total score were associated with sedentary time among people with EDSS score 4.5–6.5, but not among those with EDSS score 1.0–4.0 (Table 5). For people with EDSS score 4.5–6.5, a 1-unit increase in MFIS physical subscale and total score were associated with a 6.74 min/day (95% CI 2.11–11.37) and 2.85 min/day (95% CI 0.86–4.84) decrease in SB respectively.

**Table 5:** Sedentary time interaction analysis by EDSS subgroup.

	β	95% CI	p value
**MFIS physical**^**a**^			
EDSS 1.0–4.0	2.01	− 3.60 to 7.61	0.474
EDSS 4.5–6.5	− 6.74	− 11.37 to − 2.11	0.005
**MFIS total**^**a**^			
EDSS 1.0–4.0	0.87	− 1.78 to 3.53	0.511
EDSS 4.5–6.5	− 2.85	− 4.84 to− 0.86	0.006

^a^Adjusted for ethnicity, living arrangement, MS type.

### Prolonged bouts of sedentary time

Only the IPA social relationships subscale was associated with prolonged bouts of sedentary time (p = 0.037; Table 6). However, this association did not remain after adjusting for confounders (β = 0.67, 95% CI − 0.07, 1.42, p = 0.077).There was no evidence that associations between independent variables and prolonged bouts of sedentary time were modified by EDSS score.

**Table 6:** Associations between independent variables and prolonged bouts of sedentary time.

	Unadjusted β (95% CI)	p value	Adjusted β (95% CI)	p value
**MFIS cognitive**				
Adjusted for EDSS, employment, ethnicity, marital status, MS type	− 0.01 (− 0.06 to 0.04)	0.632	− 0.04 (− 0.09 to 0.01)	0.155
**MFIS physical**				
Adjusted for EDSS, ethnicity, marital status, MS type	− 0.01 (− 0.06 to 0.05)	0.807	− 0.05 (− 0.11 to 0.02)	0.153
**MFIS psychological**				
Adjusted for ethnicity, employment, marital status, sex	0.06 (− 0.15 to 0.28)	0.556	0.04 (− 0.21 to 0.29)	0.740
**MFIS total**				
Adjusted for EDSS, employment ethnicity, marital status, MS duration, MS type, living arrangement	0.00 (− 0.03 to 0.02)	0.766	− 0.02 (− 0.05 to 0.01)	0.179
**MSSE function**				
Adjusted for Ethnicity, MS type	0.00 (0.00 to 0.00)	0.204	0.00 (0.00 to 0.00)	0.926
**MSSE control**				
Adjusted for EDSS, ethnicity, MS type, marital status	0.00 (0.00 to 0.00)	0.871	0.00 (0.00 to 0.00)	0.174
**MSWS-12**				
Adjusted for falls history, MS type	0.01 (− 0.01 to 0.04)	0.219	0.01 (− 0.02 to 0.04)	0.529
**MSIS-29 psychological**				
Adjusted for ethnicity, MS duration, sex	0.01 (− 0.01 to 0.04)	0.207	0.01 (− 0.02 to 0.03)	0.533
**MSIS-29 physical**				
Adjusted for Ethnicity, MS type	0.01 (− 0.01 to 0.03)	0.214	0.00 (− 0.20 to 0.25)	0.828
**EQ-5D-5L utility**				
Adjusted for Ethnicity, MS duration, MS type	− 1.53 (− 3.86 to 0.80)	0.194	− 1.01 (− 3.64 to 1.63)	0.446
**IPA: autonomy indoors**				
Adjusted for ethnicity, MS type	0.39 (− 0.13 to 0.91)	0.138	0.16 (− 0.45 to 0.76)	0.601
**IPA: family role**				
Adjusted for EDSS, living arrangement, MS duration, MS type	0.11 (− 0.37 to 0.58)	0.653	− 0.06 (− 0.59 to 0.47)	0.828
**IPA: autonomy outdoors**				
Adjusted for marital status MS type	0.25 (− 0.17 to 0.67)	0.231	0.16 (− 0.29 to 0.60)	0.486
**IPA: social life and relationships**				
Adjusted for: MS Type	0.78 (0.05 to 1.50)	0.037	0.67 (− 0.07 to 1.42)	0.077

### Breaks in sedentary time

The MMSE function subscale, the MSIS-29 physical subscale, EQ-5D-5L utility score, the IPA autonomy indoors subscale and the IPA social life and relationship subscale were associated with breaks in sedentary time (Table 7). However, after adjustment for confounders, only the MMSE function subscale (β = 0.02, 95% CI 0.00, 0.04, p = 0.032), EQ-5D-5L utility score (β = 31.60, 95% CI 7.25, 55.96, p = 0.012), and IPA autonomy indoors subscale (β = − 5.11 95% CI − 9.74, − 0.48, p = 0.031) remained associated with breaks in sedentary time. Each 1-unit increase in MMSE function, indicating greater confidence in engaging in daily living activities, was associated with an additional 0.2 breaks in sedentary time/day. A 0.1 increase (i.e. improvement) in EQ-5D-5L utility score was associated with an additional 3.16 breaks/day. Each 1-unit increase in the IPA autonomy indoors subscale (i.e. worse autonomy indoors) was associated with 5.11 fewer breaks in sedentary time/day. After adjustment for MVPA, MMSE function score (β = 0.02, 95% CI − 0.01, 0.05, p = 0.122) and IPA autonomy indoors (β = − 4.44, 95% CI − 9.83, 0.96, p = 0.105) were no longer associated with breaks in sedentary time. EQ-5D-5L utility score remained associated with breaks in sedentary time after adjusting for MVPA (β = 30.17, 95% CI 4.67, 55.66, p = 0.021). There was no evidence that associations between independent variables and breaks in sedentary time were modified by EDSS score.

**Table 7:** Associations between independent variables breaks in sedentary time.

	Unadjusted β (95% CI)	p value	Adjusted β (95% CI)	p value
**MFIS cognitive**				
Adjusted for BMI, EDSS, employment, ethnicity, falls history, MS type	− 0.05 (− 0.54 to 0.44)	0.841	0.33 (− 0.21 to 0.87)	0.223
**MFIS physical**				
Adjusted for : EDSS, employment, ethnicity, falls history, MS duration, MS type, sex	− 0.15 (− 0.74 to 0.44)	0.615	0.16 (− 0.50 to 0.82)	0.627
**MFIS psychological**				
Adjusted for EDSS, employment, falls history, MS type, sex	− 0.16 (− 2.47 to 2.15)	0.889	0.96 (− 1.75 to 3.68)	0.481
**MFIS total**				
Adjusted for BMI, employment, ethnicity, MS type	− 0.05 (− 0.31 to 0.22)	0.729	0.14 (− 0.14 to 0.43)	0.313
**MSSE function**^**a**^				
Adjusted for ethnicity	0.03 (0.01 to 0.05)	0.011	0.02 (0.00 to 0.04)	0.032
**MSSE control**				
Adjusted for employment, ethnicity	0.01 (− 0.01 to 0.04)	0.260	0.00 (− 0.02 to 0.03)	0.878
**MSWS-12**				
Adjusted for EDSS, employment, ethnicity, falls history, MS duration, MS type, sex	− 0.10 (− 0.34 to 0.14)	0.411	0.02 (− 0.35 to 0.39)	0.915
**MSIS-29 psychological**				
Adjusted for : Employment, ethnicity, MS duration, MS type, sex	− 0.12 (− 0.37 to 0.13)	0.340	0.01 (− 0.27 to 0.29)	0.961
**MSIS-29 physical**				
Adjusted for Ethnicity, falls history, MS type	− 0.23 (− 0.45 to − 0.02)	0.035	− 0.16 (− 0.39 to 0.08)	0.192
**EQ-5D-5L utility**				
Adjusted for Ethnicity	36.65 (13.59 to 59.70)	0.002	31.60 (7.25 to 55.96)	0.012
**IPA: autonomy indoors**^**a**^				
Adjusted for Ethnicity, falls history	− 5.95 (− 11.32 to − 0.58)	0.030	− 5.11 (− 9.74 to − 0.48)	0.031
**IPA: family role**				
Adjusted for Employment, ethnicity, MS duration, MS type, sex	− 2.44 (− 7.46 to 2.58)	0.334	0.87 (− 4.44 to 6.18)	0.743
**IPA: autonomy outdoors**				
Adjusted for ethnicity, MS type, living arrangement	− 3.98 (− 8.33 to 0.37)	0.072	− 2.03 (− 7.01 to 2.94)	0.415
**IPA: social life and relationships**				
Adjusted for MS type, sex	− 9.62 (− 17.21 to − 2.03)	0.014	− 7.28 (− 15.35 to 0.80)	0.076

^a^Robust standard error.

## Discussion

This study quantified inclinometer measured sedentary behaviour and identified correlates in people with MS. Participants spent on average 605 min in sedentary time, had 5.9 prolonged bouts and 49.6 breaks in sedentary time per day. The control subscale of the MMSE was associated with sedentary time. The autonomy indoors subscale of the IPA, the function subscale of the MMSE and the EQ-5D-5L utility score were associated with breaks in sedentary time. No associations were demonstrated for prolonged bouts of sedentary time.

One previous study has explored sedentary behaviour outcomes in people with MS using the activPAL^23^. Participants in Manns (2020) spent on average 626.4 min in sedentary time, had 5.8 prolonged bouts and 54.6 breaks in sedentary time per day which is comparable to the present study. The average number of prolonged bouts of sedentary time are comparable to studies utilising an ActiGraph, which have shown between 4.3, and 6.1 bouts per day ^16,30,31^. Inclinometer determined sedentary time was higher than previously described self-report (range 450.9–505.6 min sitting)^13,14,18^ and count-based estimates of sedentary behaviour (range 504.5–594 min sedentary time)^16,18,30,32^. The mean number of breaks in sedentary time is also higher than previously reported accelerometer derived breaks which range from 6.9 to 14.7 ^16,30,31^ but comparable to inclinometer derived breaks in sedentary time in older adults^33,34^. Given the beneficial associations between more frequent interruptions to sedentary time and health markers^11^ quantification of sedentary breaks with a measurement tool that can robustly differentiate between sitting and standing postures is important.

In line with previous research MS type was associated with the volume and pattern of sedentary behaviour^16^. No other demographic or clinical associations were identified. This contrasts existing research which identifies age, BMI, marital status, employment status, disease duration, and disability status as correlates of self-reported sitting time and Actigraph measured SB in people with MS^13,14,16^.

Previous studies have sampled participants with mild-to-moderate mobility disability, who could walk with or without assistive devices. Differences between findings may be attributable at least in part to the different participant characteristics. Moreover, divergent measurement techniques preclude comparison. A limitation of current evidence is the use of self-report and waist worn accelerometry, which estimates SB through a lack of movement rather than postural assessment. Self-report and device-based measures of SB associate differently with health outcomes and risk^15^. Moreover, activPAL and ActiGraph measured sedentary behaviours associate differently with some health markers^35^. Accurate measurement is therefore important to determine the prevalence of SB and associated factors to target in interventions. Future studies should utilize direct assessment of sitting postures to ensure accurate measurement of sedentary behaviour.

In the present study, HRQOL and self-efficacy for function were positively associated with breaks in sedentary time. HRQOL is a multidimensional concept that examines the impact of health status on quality of life. Recent longitudinal research in the general population demonstrates a cumulative and bidirectional relationship between SB and HRQOL, implying that an action in one can result in an effect on the other in a possible virtuous cycle^36^. Indeed interventions that reduce sitting time are associated with improved HRQOL in people with MS^37^. However, targeting increases in HRQOL through interventions such as social cognitive wellness programmes ^38^ may also represent a mechanism to reduce SB among people with MS.

The positive association between self-efficacy for function (i.e. confidence in performing behaviours associated with engaging in daily living activities) and breaks in sedentary time mirrors analogous associations for self-reported and accelerometer derived sedentary time in people with MS^18^. Recent research in COPD populations demonstrates that baseline self-efficacy contributes to changes in SB^39^. Strategies to increase self-efficacy such as vicarious experience, social persuasion and performance experience of success may therefore represent important intervention strategies for sitting less and moving more. After adjusting for MVPA this association was no longer significant. Self-efficacy is positively associated with physical activity in people with MS and may have attenuated the relationship in this cohort^40^.

Enhanced feelings of control and confidence were associated with higher sedentary time in the present study. The MMSE control subscale describes an individual’s confidence to manage disease symptoms, reactions and impact on daily activities and contains items on fatigue management and activity regulation. Moreover, interaction analysis indicated higher levels of fatigue were associated with reduced time in a sitting or lying posture in participants with EDSS scores 4.5–6. Sitting represents a commonly used energy conservation strategy in this population where fatigue is a persistent and highly debilitating issue^41,42^. Collectively these results highlight the potential value of utilising sedentary time as a resting or pacing mechanism to control symptoms and reduce fatigue. However, excessive sedentariness may aggravate disease and SB comorbidities in the long term. Future interventions should promote sedentary modification while acknowledging the value of rest and pacing for fatigue and symptom management.

The IPA autonomy indoors subscale which explores the ability to look after oneself and get around the house as wanted was associated with less breaks in sedentary time. Autonomy indoors correlates with physical function ^43,44^ and activities of daily living (ADLs) performance^45^. Accordingly, low autonomy over self-care and the home environment may limit opportunities to move more and sit less resulting in sedentary time accumulation. Limited research has identified environmental factors ^46^ and appraisal (i.e. a positive view of situations and the ability to deal with them) ^47^ as predictors of participation and autonomy among people with MS. Consideration of environmental barriers and their impact on perceived participation and incorporation of interventions that foster positive appraisal, coping styles and empowerment may represent potential strategies to enhance autonomy for performance of ADLs with corresponding benefits to SB.

The cross-sectional study design precludes any inferences of causality between SB and independent variables. Participants were community dwelling, mostly female, and white. Results therefore are not necessarily generalizable to the wider population of people with MS. Furthermore, data is drawn from a self-selecting sample from a behaviour change intervention. It is possible that the sample was biased towards those already engaged in activity or conversely to those who were inactive, which may have impacted the baseline sedentary data.

Understanding of the determinants of sedentary time and pattern may inform future interventions for reducing SB. This study represents an initial step towards classifying modifiable correlates of sedentary time and patterns. Based on our findings, interventions targeting reductions in SB should consider strategies that enhance self-efficacy, foster participation and autonomy and improve perceived health related quality of life domains.

## Supplementary Information

Supplementary Tables.

## References

[1] Tremblay, M. S. *et al.* Sedentary behavior research network (SBRN)–terminology consensus project process and outcome. *Int. J. Behav. Nutr. Phys. Act.* **14**, 75. 10.1186/s12966-017-0525-8 (2017). 10.1186/s12966-017-0525-8 PMC5466781 28599680

[2] Katzmarzyk PT (2019). Sedentary behavior and health: Update from the 2018 Physical Activity Guidelines Advisory Committee. Med. Sci. Sports Exerc..

[3] Veldhuijzen van Zanten JJ, Pilutti LA, Duda JL, Motl RW (2016). Sedentary behaviour in people with multiple sclerosis: Is it time to stand up against MS?. Multiple Sclerosis (Houndmills, Basingstoke, England).

[4] Hubbard EA, Motl RW (2015). Sedentary behavior is associated with disability status and walking performance, but not cognitive function, in multiple sclerosis. Appl. Physiol. Nutr. Metab..

[5] Hubbard EA, Motl RW, Fernhall B (2018). Sedentary behavior and blood pressure in patients with multiple sclerosis. Int. J. MS Care.

[6] Freeman JA (2016). Standing up in multiple sclerosis (SUMS): Protocol for a multi-centre randomised controlled trial evaluating the clinical and cost effectiveness of a home-based self-management standing frame programme in people with progressive multiple sclerosis. BMC Neurol..

[7] Saunders, T. J. *et al.* Sedentary behaviour and health in adults: An overview of systematic reviews. **45**, S197–S217. 10.1139/apnm-2020-0272 (2020). 10.1139/apnm-2020-0272 33054341

[8] Diaz KM (2017). Patterns of sedentary behavior and mortality in U.S. middle-aged and older adults: A national cohort study. Ann. Internal Med..

[9] Healy GN (2008). Breaks in sedentary time: Beneficial associations with metabolic risk. Diabetes Care.

[10] Jefferis BJ (2015). Duration and breaks in sedentary behaviour: Accelerometer data from 1566 community-dwelling older men (British Regional Heart Study). Br. J. Sports Med..

[11] Chastin SF, Egerton T, Leask C, Stamatakis E (2015). Meta-analysis of the relationship between breaks in sedentary behavior and cardiometabolic health. Obesity (Silver Spring, Md.).

[12] Bellettiere J (2017). Associations of sitting accumulation patterns with cardio-metabolic risk biomarkers in Australian adults. PLoS ONE.

[13] Sasaki JE (2018). National estimates of self-reported sitting time in adults with multiple sclerosis. Multiple Sclerosis J. Exp. Transl. Clin..

[14] Hubbard EA, Motl RW, Manns PJ (2015). The descriptive epidemiology of daily sitting time as a sedentary behavior in multiple sclerosis. Disabil. Health J..

[15] Prince SA (2020). A comparison of self-reported and device measured sedentary behaviour in adults: A systematic review and meta-analysis. Int. J. Behav. Nutr. Phys. Act..

[16] Jeng B, Sasaki JE, Cederberg KL, Motl RW (2019). Sociodemographic and clinical correlates of device-measured sedentary behaviour in multiple sclerosis. Disabil. Rehabil..

[17] Neal WN, Cederberg KL, Jeng B, Sasaki JE, Motl RW (2020). Is Symptomatic fatigue associated with physical activity and sedentary behaviors among persons with multiple sclerosis?. Neurorehabil. Neural Repair..

[18] Motl RW, Sasaki JE, Cederberg KL, Jeng B (2019). Social-cognitive theory variables as correlates of sedentary behavior in multiple sclerosis: Preliminary evidence. Disabil. Health J..

[19] Janssen X, Cliff DP (2015). Issues related to measuring and interpreting objectively measured sedentary behavior data. Meas. Phys. Educ. Exerc. Sci..

[20] Tudor-Locke C, Johnson WD, Katzmarzyk PT (2011). US population profile of time-stamped accelerometer outputs: Impact of wear time. J. Phys. Act. Health.

[21] Sellers C, Dall P, Grant M, Stansfield B (2016). Validity and reliability of the activPAL3 for measuring posture and stepping in adults and young people. Gait Posture.

[22] Kozey-Keadle, S. *et al.* Validation of wearable monitors for assessing sedentary behavior. *Med. Sci. Sports Exerc*. **43**, 1561–1567. 10.1249/MSS.0b013e31820ce174 (2011). 10.1249/MSS.0b013e31820ce174 21233777

[23] Manns PJ, Mehrabani G, Norton S, Aminian S, Motl RW (2020). The SitLess with MS program: Intervention feasibility and change in sedentary behavior. Arch. Rehabilit. Res. Clin. Transl..

[24] Ryan JM (2019). Safety, feasibility, acceptability and effects of a behaviour-change intervention to change physical activity behaviour among people with multiple sclerosis: Results from the iStep-MS randomised controlled trial. Multiple Sclerosis (Houndmills, Basingstoke, England).

[25] Winkler EA (2016). Identifying adults' valid waking wear time by automated estimation in activPAL data collected with a 24 h wear protocol. Physiol. Meas..

[26] Prescott S (2020). Minimum accelerometer wear-time for reliable estimates of physical activity and sedentary behaviour of people receiving haemodialysis. BMC Nephrol..

[27] van Hout B (2012). Interim scoring for the EQ-5D-5L: Mapping the EQ-5D-5L to EQ-5D-3L value sets. Value Health J. Int. Soc. Pharmacoecon. Outcomes Res..

[28] Ryan JM (2017). Changing physical activity behaviour for people with multiple sclerosis: protocol of a randomised controlled feasibility trial (iStep-MS). BMJ Open.

[29] Biddle SJH (2019). Controversies in the science of sedentary behaviour and health: Insights, perspectives and future directions from the 2018 Queensland Sedentary Behaviour Think Tank. Int. J. Environ. Res. Public Health.

[30] Ezeugwu V, Klaren RE, Hubbard EA, Manns PT, Motl RW (2015). Mobility disability and the pattern of accelerometer-derived sedentary and physical activity behaviors in people with multiple sclerosis. Prevent. Med. Rep..

[31] Cederberg KLJ (2019). Physical activity, sedentary behavior, and restless legs syndrome in persons with multiple sclerosis. J. Neurol. Sci..

[32] Klaren RE (2015). Objectively measured physical activity is associated with brain volumetric measurements in multiple sclerosis. Behav. Neurol..

[33] Čukić I (2019). Cross-sectional associations between personality traits and device-based measures of step count and sedentary behaviour in older age: The Lothian Birth Cohort 1936. BMC Geriatr..

[34] Okely JA (2019). Positive and negative well-being and objectively measured sedentary behaviour in older adults: Evidence from three cohorts. BMC Geriatr..

[35] Edwardson CL (2020). activPAL and ActiGraph assessed sedentary behavior and cardiometabolic health markers. Med. Sci. Sports Exerc..

[36] Omorou AY (2016). 10-year cumulative and bidirectional associations of domain-specific physical activity and sedentary behaviour with health-related quality of life in French adults: Results from the SU.VI.MAX studies. Prevent. Med..

[37] McAuley E (2015). Effects of a DVD-delivered exercise intervention on physical function in older adults with multiple sclerosis: A pilot randomized controlled trial. Multiple Sclerosis J. Exp. Translat. Clin..

[38] Jongen PJ (2014). Improved self-efficacy in persons with relapsing remitting multiple sclerosis after an intensive social cognitive wellness program with participation of support partners: A 6-months observational study. Health Qual. Life Outcomes.

[39] Liacos A (2019). The Pulmonary Rehabilitation Adapted Index of Self-Efficacy (PRAISE) tool predicts reduction in sedentary time following pulmonary rehabilitation in people with chronic obstructive pulmonary disease (COPD). Physiotherapy.

[40] Motl RW, Snook EM (2008). Physical activity, self-efficacy, and quality of life in multiple sclerosis. Ann. Behav. Med..

[41] Blikman LJ (2013). Effectiveness of energy conservation treatment in reducing fatigue in multiple sclerosis: A systematic review and meta-analysis. Arch. Phys. Med. Rehabil..

[42] Aminian S, Ezeugwu VE, Motl RW, Manns PJ (2019). Sit less and move more: Perspectives of adults with multiple sclerosis. Disabil. Rehabil..

[43] Ryan JM, Stennett AM, Peacock S, Baker G, Norris M (2019). Associations between activity and participation in adults with multiple sclerosis: a cross sectional study. Physiotherapy.

[44] Chen X (2018). Perceived participation and its correlates among first-stroke survivors at six months after discharge from a tertiary hospital in China. Arch. Phys. Med. Rehabil..

[45] Chen X, He Y, Meng X, Zhou L (2017). Factors associated with perceived participation three months after being discharged from a tertiary hospital. Clin. Rehabil..

[46] Karhula ME (2019). Predictors of participation and autonomy in people with multiple sclerosis. Am. J. Occup. Ther..

[47] van den Akker LE (2016). The role of appraisal and coping style in relation with societal participation in fatigued patients with multiple sclerosis: A cross-sectional multiple mediator analysis. J. Behav. Med..

