# Supplementary material for: Patterns and correlates of sedentary behaviour among people with multiple sclerosis: a cross-sectional study
Source: Sci Rep. 2021 Oct 13;11:20346. doi: 10.1038/s41598-021-99631-z (PMC8514488; doi:10.1038/s41598-021-99631-z)
Supplement: Supplementary file 1 — Supplementary Tables. [file 41598_2021_99631_MOESM1_ESM.pdf]

Supplementary table 1: Variable scoring

Name	Purpose	Structure	Min-max
MFIS cognitive subscale	Impact of fatigue on cognitive activities	10 items (0-4)	0-40 (higher = more fatigue impact)
MFIS physical subscale	Impact of fatigue on physical activities	9 items (0-4)	0 to 36 (higher = more fatigue impact)
MFIS psychosocial subscale	Impact of fatigue on physical activities	2 items (0-4)	0-8 (higher = more fatigue impact)
MFIS total score	Impact of fatigue on a person's activities	21 items (0-4)	0-84 (higher = more fatigue impact)
MMSE control subscale	Confidence with managing symptoms and coping with demands of illness	9 items (10-100)	90-900 (higher = more confidence)
MMSE function subscale	Confidence with regard to functional abilities	9 items (10-100)	90-900 (higher = more confidence)
MSWS-12 total score	Impact of MS on a person's walking capability over the past 2week	12 items (1-5)	12-60 converted to percentage (higher % = poorer walking capability)
MSIS-29 physical	Physical impact of MS	20 items (1-5)	(0-100) higher = indicates greater impact of disease on daily function
MSIS-29 psychological	Psychological impact of MS	9 items (1-5)	(0-100) higher = indicates greater impact of disease on daily function
EQ5DL index	Health state	5 items (1-5)	Higher = better health state
IPA: autonomy indoors	Participant and autonomy indoors	7 items (0-4)	0-28 (higher = worse autonomy and participation)
IPA: family role	Family participation and autonomy	7 items (0-4)	0-28 (higher = worse autonomy and participation)
IPA: autonomy outdoors	Participation and autonomy outdoors	5 items (0-4)	0-20 (higher = worse autonomy and participation)
IPA: social life and relationships	Social participation and autonomy	7 items (0-4)	0-28 (higher = worse autonomy and participation)

Supplementary table 2: Sedentary outcomes based on demographic and clinical characteristics

	Average sedentary time, (min/day)	Average prolonged bouts (≥30 min) of sitting/lying (n/day)	Average number of breaks in sitting per day (n/day)
	Mean (SD)	Mean (SD)	Mean (SD)
Sex			
Male	608.63 (109.76)	6.13 (1.85)	43.76 (16.43)
Female	602.33 (107.86)	5.79 (1.59)	52.58 (17.83)
Ethnicity			
White	596.81 (107.63)	5.83 (1.66)	51.06 (17.57)
Black	638.45 (108.81)	6.05 (1.31)	45.27 (2.54)
Asian	698.05 (78.19)	7.05 (2.33)	29.32 (19.28)
Living arrangement			
Lives alone	645.04 (128.76)	6.41 (2.19)	49.04 (9.18)
Lives with partner/spouse/family member	599.60 (105.19)	5.84 (1.62)	49.65 (18.55)
Employment status			
Employed	598.72 (115.82)	5.72 (1.49)	53.31 (14.36)
Not employed	607.42 (104.58)	6.00 (1.77)	47.67 (19.12)
Marital status			
Married/partnered	600.11 (110.27)	5.82 (1.66)	49.85 (19.37)
Not married/partnered	622.31 (98.36)	6.24 (1.77)	48.51 (8.69)
Type of MS			
Relapsing-remitting	551.41 (106.69)	5.29 (2.02)	54.42 (19.83)
Secondary progressive	643.95 (112.92)	6.43 (1.39)	43.06 (15.11)
Primary progressive	629.19 (83.06)	5.93 (1.46)	51.70 (19.19)
Unknown	578.75 (60.46)	6.13 (1.30)	52.33 (4.45)
EDSS			
1.0-4.0	581.01 (88.03)	5.64 (1.19)	53.62 (14.24)
4.5-6.5	613.05 (113.62)	6.00 (1.82)	48.11 (18.78)

MS: multiple sclerosis; EDSS: Expanded Disability Status Scale; SD: standard deviation
